# Translation, cross-cultural adaptation, and psychometric properties of the family impact scale: a COSMIN-based systematic review

**DOI:** 10.1186/s12955-025-02473-w

**Published:** 2025-12-30

**Authors:** Patcharamon Kunsavate, Warit Powcharoen, Areerat Nirunsittirat, Chanika Manmontri

**Affiliations:** 1 Division of Pediatric Dentistry, Department of Orthodontics and Pediatric Dentistry, Faculty of Dentistry, Chiang Mai University, Suthep Rd., Mueang, Chiang Mai, 50100 Thailand; 2 Department of Oral and Maxillofacial Surgery, Faculty of Dentistry, Chiang Mai University, Chiang Mai, Thailand; 3 Division of Dental Public Health, Department Advanced General Dentistry and Dental Public Health, Faculty of Dentistry, Chiang Mai University, Chiang Mai, Thailand

**Keywords:** Family impact scale, Oral health-related quality of life, Psychometric properties, Cross-cultural adaptation, COSMIN, Pediatric oral health, Caregiver burden

## Abstract

**Background:**

The Family Impact Scale (FIS) is a validated caregiver-reported outcome measure that gauges the impact of children’s oral and orofacial conditions on their families. As its use continues to expand across countries and cultures, the quality of its translated versions and their psychometric properties must be rigorously evaluated. This systematic review sought to assess the translations, cross-cultural adaptations and measurement properties of FIS-14 and FIS-8.

**Methods:**

This review followed PRISMA 2020 guidelines and the COSMIN methodology. PubMed, Scopus, and Embase were searched, with studies being eligible if they translated or adapted the FIS and evaluated at least one psychometric property among caregivers of children aged 6–14 years. Data extraction and quality appraisal were performed independently by two reviewers. Translation and adaptation processes were evaluated using the framework for translation and cultural adaptation process for patient-reported outcomes measures. Psychometric properties and risk of bias were assessed using COSMIN criteria.

**Results:**

Fourteen studies met the inclusion criteria, including three studies utilising FIS-8, ten studies utilising the FIS-14 and one study using both versions, covering nine different language adaptations in total. Most studies adhered to core translation and adaptation steps, but key methodological elements, such as reconciliation, cognitive debriefing and proofreading, were frequently inadequately reported. Content validity was frequently rated “doubtful” due to a lack of methodological clarity. The internal consistency of FIS-14 was acceptable (FIS-14: α = 0.79–0.88), but FIS-8 exhibited inconsistent results. Test–retest reliability was generally good (ICC = 0.75–0.96) but often lacked confidence intervals or model specification. Construct validity was supported in most studies. Measurement error and responsiveness were underreported. Structural validity was only assessed in two studies, while cross-cultural and criterion validity were not evaluated in any studies.

**Conclusions:**

FIS-14 demonstrates acceptable reliability and construct validity across a diverse range of settings, supporting its use in assessments of oral health-related quality of life. However, substantial methodological gaps persist when it comes to content validation, responsiveness, structural validity and cross-cultural testing. Future adaptations should follow established guidelines and employ robust statistical methods to ensure comparability and interpretability across different populations.

**Supplementary Information:**

The online version contains supplementary material available at 10.1186/s12955-025-02473-w.

## Introduction

Oral health–related quality of life (OHRQoL) instruments have emerged as essential complements to traditional clinical and epidemiological indicators. While conventional indices quantify disease burden, they fail to capture the broader functional, psychological and social consequences of oral conditions. The introduction of socio-dental indicators [1] highlights the need to integrate subjective experiences and psychosocial impacts into oral health assessments. This shift in perspective spurred the development of instruments geared towards evaluating OHRQoL among both adults and children.

Increasing attention has also been directed to the family dimension of OHRQoL, recognising that a child’s oral health problems can disrupt household routines, emotional climate, and financial stability. Recent evidence demonstrates that childhood oral diseases impose substantial emotional strain, caregiving burden, and economic costs on families, for example among those caring for children with orofacial clefts, [2] early childhood caries, [3] or developmental conditions such as autism spectrum disorder [4]. These findings highlight the importance of assessing family impact as a distinct construct within pediatric oral health research and underscore the need for instruments that are culturally adaptable, valid, and reliable across diverse populations.

Within pediatric dentistry, instruments such as the Child Oral Health Quality of Life Instrument (COHQOL) and the Early Childhood Oral Health Impact Scale (ECOHIS) incorporate domains assessing caregiver and family burdens [5, 6]. The ECOHIS includes a dedicated family domain,[6] whereas the COHQOL integrates the Family Impact Scale (FIS) within the Parental–Caregiver Perceptions Questionnaire (P-CPQ) [7]. These instruments highlight the importance of capturing family-level outcomes in OHRQoL assessment.

The FIS was developed by Locker et al. in 2002, [5] consisting of 14 items across four subdomains, including Parental/Family Activity, Parental Emotions, Family Conflict and Financial Burden, and is to be completed by the child’s primary caregiver. Although originally part of the P-CPQ, the FIS has since been used independently in numerous studies because of its conceptual clarity and practical utility [8–10]. The responses reflect caregiver-reported family impact, a construct distinct from self-reported (by the child) information or information on the caregiver’s own quality of life. A shortened eight-item version (FIS-8) was introduced in 2013 to improve its feasibility in research and clinical settings, retaining the original’s conceptual framework while reducing the number of items to eight across three domains: Parental Activity, Parental Emotions and Family Conflict [11]. Both versions acknowledge that chronic oral conditions affect not only the child but also family dynamics, routines, emotional well-being, and financial strain [12]. 

Because perceptions of child health, caregiving norms, and family functioning vary across cultural settings, rigorous translation and cultural adaptation of the FIS-14 and FIS-8 are essential to ensure conceptual equivalence [13, 14]. The COnsensus-based Standards for the Selection of Health Measurement INstruments (COSMIN) framework provides internationally recognised methodological standards for evaluating the quality of translation, adaptation, and psychometric properties [15]. COSMIN highlights key domains including content validity, structural validity, internal consistency, cross-cultural validity, reliability, measurement error, criterion validity, construct validity, and responsiveness, thereby ensuring that translated instruments retain conceptual clarity and measurement adequacy [15]. 

Best-practice guidelines describe core steps in translation and cross-cultural adaptation including forward translation, backward translation, expert committee review, and cognitive pre-testing to ensure comprehensibility and cultural relevance of the translated version [13, 14]. Following adaptation, psychometric evaluation is necessary to determine whether the translated instrument preserves validity, reliability, and conceptual integrity in the new context [14, 15]. 

Although several versions of the FIS have been translated and adapted for different cultural settings, many were developed before the widespread adoption of COSMIN standards, [15] resulting in considerable variability in methodological quality. To date, no systematic review has synthesised the translation procedures and psychometric properties of FIS-14 and FIS-8 using COSMIN criteria. This gap limits the ability of clinicians and researchers to identify high-quality, culturally appropriate versions. Therefore, this systematic review aims to identify, evaluate, and synthesise 1) the methodological rigour and transparency of translation and cultural adaptation procedures, and (2) the psychometric properties reported across studies according to COSMIN.

## Materials and methods

### Study design and registration

This systematic review adhered to the PRISMA (Preferred Reporting Items for Systematic Reviews and Meta-Analyses) 2020 guidelines [16] and followed the COSMIN guideline for systematic reviews of patient-reported outcome measures version 2.0 [15]. The protocol was registered prospectively in the International Prospective Register of Systematic Reviews (PROSPERO, CRD42024592315) [17]. 

### Eligibility criteria

*Inclusion criteria*:

- Studies that involved the translation, cross-cultural adaptation, and/or psychometric evaluation of the FIS.
- Studies that reported at least one psychometric property, including internal consistency, test–retest reliability, content validity, structural validity, cross-cultural validity, measurement error, hypothesis testing for construct validity, or responsiveness.
- Studies that used either the full (FIS-14) or the shortened version (FIS-8).
- Studies that assessed caregivers of children aged 6–14 years with oral health conditions (e.g., caries, malocclusion, oral hygiene status).
- Studies that were published in English.

*Exclusion criteria*:

- Studies that used the FIS solely as an outcome measure without detailing adaptation or validation procedures.
- Studies involving preschool-aged children, adult populations, or caregivers outside the intended 6–14 age range.
- Studies evaluating unrelated OHRQoL instruments.
- Reviews, conference abstracts, dissertations, case reports, and qualitative studies without psychometric evaluation.

### Search strategy

A comprehensive search was performed in PubMed, Embase (Ovid), and Scopus from 1 January 2002 (year of FIS development) to 31 March 2025. The final search was conducted on 31 March 2025. Only English-language publications were included, and no restrictions were placed on study design.

Search strategies combined controlled vocabulary (e.g., MeSH, Emtree) and free-text terms related to the FIS (e.g., “family impact questionnaire”, “FIS”) and psychometric measurement properties (e.g., “validity”, “reliability”, “internal consistency”, “cross-cultural adaptation”, “psychometrics”). Boolean operators (AND/OR/NOT), and field-specific syntax were used to optimise sensitivity and specificity. Variants and abbreviations of the FIS were also included. Exclusion terms (e.g., “Fatigue Impact Scale”, “Functional Independence Scale”) were incorporated to minimise retrieval of unrelated instruments. The database search was performed by one reviewer (CM), and search terms and results were verified by the review team. Full search strategies for each database are available in Supplementary Appendix 1.

All retrieved records were imported into Rayyan [18] for de-duplication and screening. Reference lists of included studies and relevant reviews were screened manually to identify additional records.

### Study selection and data extraction

Two reviewers (WP and AN) independently screened titles and abstracts using Rayyan [18]. Full-texts were assessed for eligibility. Disagreements were resolved through discussion, or by consulting a third reviewer (CM) when necessary. Data extraction was performed independently by two reviewers (CM and PK) using a structured form developed according to the COSMIN guidelines [15]. Extracted data included:

- Study characteristics: authors, year, country, setting, language.
- Population characteristics: caregiver demographics, sample size, child age, child’s oral condition, FIS version.
- Translation and cross-cultural adaptation processes [14].
- Psychometric properties assessed [15].
- FIS results: mean scores, floor/ceiling effects, subdomain patterns, subgroup analyses.

Extracted data were compared, and discrepancies were resolved through consensus.

### Evaluation of translation and cross-cultural adaptation

Translation and adaptation procedures were assessed using a structured rubric based on Wild et al. (2005), [14] which evaluates eight steps of good practice. Each step was rated as fully adherent (+), partially adherent (±), not adherent (−) or unclear (?). Two reviewers (CM and PC) conducted these assessments independently, with disagreements resolved by discussion or adjudication by a third reviewer (WP). The complete rubric is available in Supplementary Appendix 2.

### Evaluation of psychometric properties

Psychometric properties were assessed following the COSMIN guideline for systematic reviews of patient-reported outcome measures, version 2.0 [15]. The following nine properties were assessed when reported: content validity, structural validity, internal consistency, cross-cultural validity/measurement invariance, reliability, measurement error, criterion validity, hypothesis testing for construct validity, and responsiveness. Each property was graded as sufficient (+), insufficient (−), indeterminate (?), or not assessed (NA) using the COSMIN criteria for good measurement properties (version 2.0) [15]. Floor/ceiling effects were considered present when more than 15% of respondents achieved the minimum or maximum score. Evaluations were performed independently by two reviewers (CM and PC), with discrepancies resolved by discussion or third-reviewer adjudication (WP).

### Evaluation of the methodological quality and risk of bias assessment

Methodological quality was assessed using the COSMIN Risk of Bias Checklist, version 3 [15]. This checklist evaluates study design, statistical methods, and reporting for each psychometric property. In accordance with COSMIN’s “worst score counts” principle, the lowest item rating within each property determined its overall level of risk of bias as very good (VG), adequate (A), doubtful (D), or inadequate (IA) [15]. Two reviewers (WP and AN) performed assessments independently; disagreements were resolved through team discussion (CM and PK).

Due to substantial heterogeneity across studies in language versions, methodologies, and psychometric analyses, a narrative synthesis was conducted rather than a meta-analysis.

## Results

### Search result

**Fig. 1:**
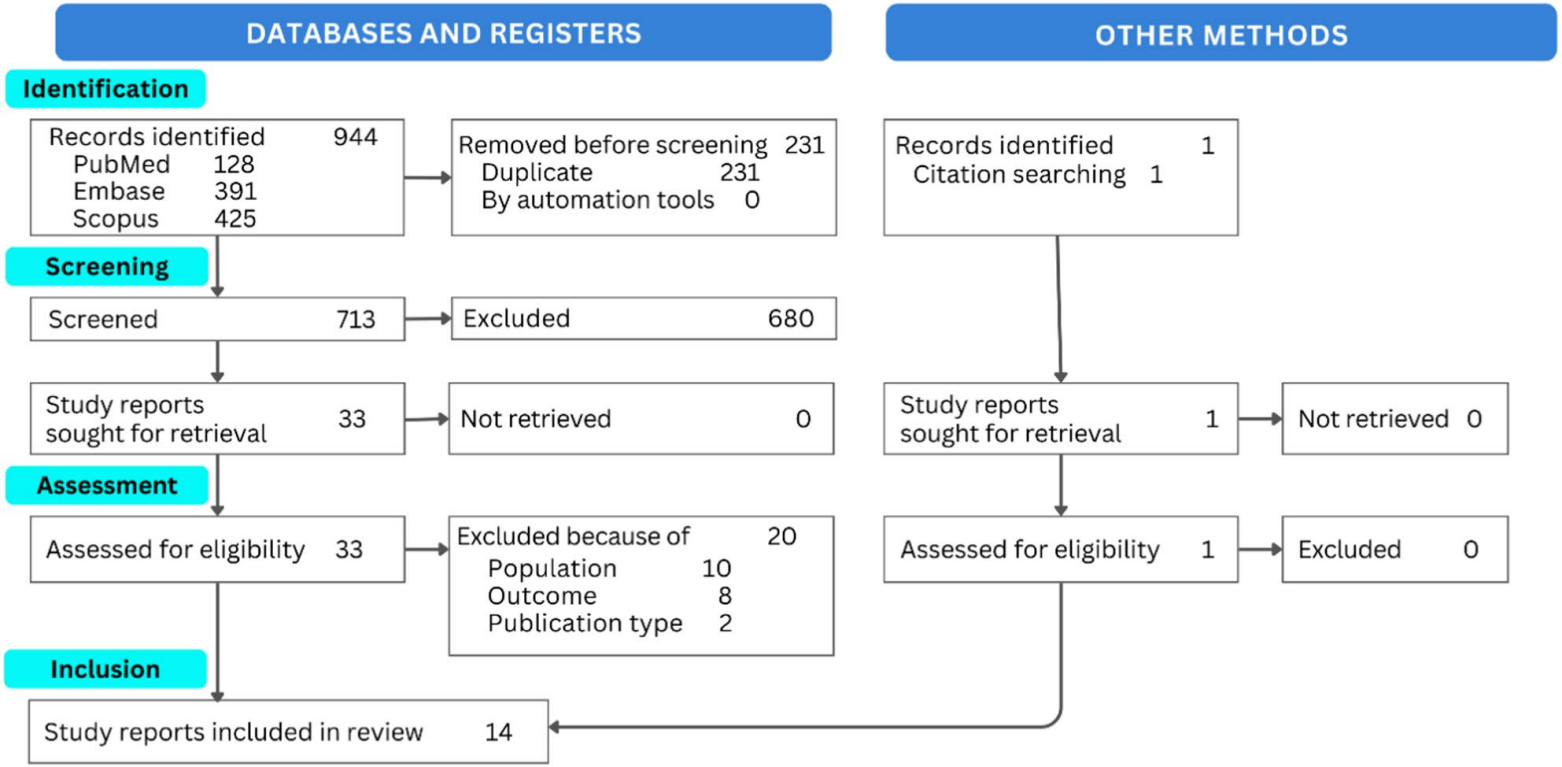
PRISMA flow of the study-selection process

A total of 944 articles were identified from three databases (PubMed = 128, Embase = 391, Scopus = 425). After removing 231 duplicates, 713 unique records proceeded to title and abstract screening, during which 680 were excluded for not meeting the inclusion criteria. A total of 33 full-text articles were retrieved for assessment, of which 20 were excluded due to ineligible population (*n* = 10), ineligible outcome (*n* = 8), or ineligible publication type (*n* = 2). The complete list of excluded articles and reasons for exclusion is provided in Supplementary Appendix 3. One additional record was identified through citation searching and assessed in full, yielding no exclusions. Ultimately, 14 studies fulfilled all eligibility criteria and were included in this review. The full study selection pathway is presented in Fig. 1.

### Characteristics of included studies

The 14 included studies were published between 2002 and 2024 and were conducted across 10 countries, producing nine language versions of the FIS: English (Canada, United Kingdom), [5, 19, 20] Arabic (Oman, Saudi Arabia, Libya), [21–23] Brazilian Portuguese (Brazil), [24, 25] Cantonese (Hong Kong), [26] Croatian (Croatia), [27] Hindi (india), [28] Kannada (India), [29] Telugu (India), [30] and Peruvian Spanish (Peru) [31]. The majority of studies used the FIS-14 (*n* = 10) [19, 20, 22, 24–29, 31], while the FIS-8 was used in four studies [21, 23, 27, 30]. One study evaluated both the FIS-14 and the FIS-8 in the same population [27]. Table 1 summarises the key characteristics of each study.

**Table 1:** Characteristics of included studies

Study	Country (Language)	Cross-Sectional Design	Setting	Number of Parents or Caregivers	Children’s Age Range (Years)	Oral Health Conditions Studied				FIS Version
						Dental Caries	Gingival Health	Mal-occlusion	Other Conditions	
Locker, 2022 [5] (Original)	Canada (English)	Yes	Clinical & academic	93	6–14	Yes	Yes	Yes	Dental trauma, cleft, craniofacial anomalies, amelogenesis imperfecta	FIS-14
Marshman, 2007 [19]	United Kingdom (English)	Yes	Clinical & academic	87	8–14	Yes	Yes	Yes	Enamel opacities	FIS-14
Agou, 2008 [20]	Canada (English)	No, cohort	Academic	26	11–14	-	-	Yes	-	FIS-14
Al-Riyami, 2016 [21]	Oman (Arabic)	Yes	Military dental clinic	191	2–9	-	-	-	-	FIS-8
Quadri, 2021 [22]	Saudi Arabia (Arabic)	Yes	Online	500	Primary school	-	Yes	-	-	FIS-14
Mansur, 2022 [23]	Libya (Arabic)	Yes	Community	246	12	Yes	Yes	-	-	FIS-8
Goursand, 2009 [24]	Brazil (Portuguese)	Yes	Academic	123	11–14	Yes	-	Yes	-	FIS-14
Barbosa, 2009 [25]	Brazil (Portuguese)	Yes	Community	210	8–14	Yes	-	Yes	-	FIS-14
McGrath, 2007 [26]	Hong Kong (Cantonese)	Yes	Academic	168	11–14	Yes	-	Yes	-	FIS-14
Pipovic, 2024 [27]	Croatia (Croatian)	Mainly, subset cohort	Academic	193	11–14	Yes	-	Yes	-	FIS-14, FIS-8
Purohit, 2021 [28]	India (Hindi)	Yes	Community	225	12–15	Yes	Yes	-	-	FIS-14
Vinayagamoorthy, 2020 [29]	India (Kannada)	Yes	Community	768	12–15	Yes	-	Yes	-	FIS-14
Kumar, 2016 [30]	India (Telugu)	Yes	Community	1,342	11–14	Yes	-	Yes	Fluorosis	FIS-8
Abanto, 2015 [31]	Peru (Spanish)	Yes	Community	200	11–14	Yes	-	Yes	-	FIS-14

FIS = Family Impact Scale

Of the 14 included studies, 13 were cross-sectional in design [5, 19, 21–31]. The Canadian study [20] was the only study conducted entirely as a cohort to evaluate responsiveness. The Croatian version [27] was primarily cross-sectional, but a subset of 46 parents was followed longitudinally before and after their children’s one-year orthodontic treatment to assess responsiveness. Seven studies were conducted in clinical or academic settings (e.g., pediatric dental clinics, orthodontic dental clinics), [5, 19–21, 24, 26, 27] while six were community-based, typically through schools [23, 25, 28–31]. One study on the Saudi Arabic version [22] collected data through an online survey without conducting any clinical oral examinations, contrasting with the other studies, which entailed clinical assessments by calibrated examiners.

Most studies used self-administered questionnaires, either in clinical settings, [5, 19–21, 24, 26, 27] at home [22] or at school [25, 28–31]. Only one study used interview-based administration [31]. Sampling approaches varied: random sampling or cluster sampling in some community-based studies, [23, 28–30] convenience sampling in most clinical studies, [5, 19–22, 26, 27] and unspecified sampling in a few reports [22, 25]. 

### Participant characteristics

All studies specified their inclusion criteria, which commonly involved children aged 6–14 years with no systemic or developmental disorders and caregivers’ capable of understanding the questionnaire language [5, 20, 22, 23, 25–31]. Eight studies explicitly reported exclusion criteria, [5, 19–21, 25, 28–30] while six did not [22–24, 26, 27, 31]. Reported exclusion criteria included incomplete caregiver responses, [5, 19] child-related medical or developmental conditions (e.g., craniofacial anomalies, cognitive or behavioral impairments), [20, 21, 29] caregiver illiteracy, [21, 25] children’s inability to undergo an oral examination [25, 28, 30] and children with a prior or ongoing orthodontic treatment [29]. 

In the phase assessing psychometric properties, caregiver response rates were generally high (76.9%–96%) [21, 23–31]. Sample sizes ranged from 26 to 1,342 caregivers, with children’s age spanning 6–15 years, though some studies targeted narrower age groups (e.g., solely 12-year-olds, [23] 12–15-year-olds, [28, 29] and 11–14-year-olds [24, 26, 27, 30, 31]). One exception was the Arabic version tested in Oman, [21] which involved caregivers of children outside the target age range (2–9 years). Mothers were the primary respondents among the studies that reported this information, accounting for 54.5–90.2% of participants [5, 19, 23–25, 27, 30]. Of the 14 studies, seven specified caregivers’ roles, [5, 19, 23–25, 27, 30] and five reported caregiver socioeconomic indicators such as education and employment [21, 23, 25, 29, 30]. 

Children’s conditions included dental caries (most often using WHO criteria) [5, 19, 24–26, 28−31]; malocclusion (measured by the Dental Aesthetic Index or the Index of Orthodontic Treatment Need) [5, 19, 20, 25–27, 29−31]; parents perceived oral hygiene status, [27] gingivitis [19, 23, 28]; and fluorosis [30]. Some studies focused on specific populations, such as children undergoing orthodontic treatment [20] and those with early childhood caries, [21] while others evaluated school-based samples across diverse socioeconomic backgrounds [22, 25, 28–31]. 

### Translation and cross-cultural adaptation of the included studies

Eleven studies reported translation and adaptation procedures for FIS-14 or FIS-8 across eight countries. A summary of the translation and cross-cultural adaptation processes using a structured rubric based on Wild et al. (2005), [14] is presented in Table 2. *Forward translation* was performed in most studies [21, 22, 24–31]; however, the number and qualifications of translators varied. Only four versions (Brazilian Portuguese, [24, 25] Croatian, [27] Peruvian Spanish [31]) used two independent forward translators fluent in both the source and target languages, as recommended. Several studies used only one translator [22, 29] or lacked information on translator qualifications [21, 26, 28, 30]. *Reconciliation* (i.e., the resolution of translation discrepancies) was clearly described in four studies, [25, 27, 30, 31] partially reported in four, [21, 22, 28, 29] and not reported in three [23, 24, 26]. 

*Back-translation* was fully described in four versions [24, 25, 27, 31] and partially reported in others without the specification of translators’ qualifications [21, 22, 26, 28–30]. While most studies performed the step of *review and harmonization* (compared the back-translated version to the original), [21, 22, 24–27, 29−31] a few did not provide sufficient information to determine whether this step had been conducted [23, 28]. 

*Cognitive debriefing* (pilot testing) was reported in most studies, [21, 25–31] although sample sizes and representativeness were inconsistent. Three studies provided insufficient information [24] or omitted this step [22, 23]. Modifications following cognitive debriefing (*review and finalisation*) were often reported but lacked detail [21, 22, 25, 26, 28–31]. Only the Croatian version [27] clearly stated that no changes were made, as a panel of four experts finalised the instrument [27]. *Proofreading* and documentation of the *final adapted version* were not reported in any study. These gaps indicate variable adherence to recommended guidelines for cross-cultural adaptation.

**Table 2:** Assessment of the translation and cross-cultural adaptation process among the included studies according to the principles of good practice for the translation and cross-cultural adaptation process for patient-reported outcome measures

Study	Country (language)	Forward Translation	Reconci-liation	Back Translation	Review and Harmo-nisation	Cognitive Debriefing	Review and Finali-sation	Proof-reading	Final Report
Al-Riyami, 2016 [21]	Oman (Arabic)	±	–	±	±	±	±	?	±
Quadri, 202 [22]	Saudi Arabia (Arabic)	±	–	±	±	–	±	?	±
Mansur, 2022 [23]	Libya (Arabic)	?	?	?	?	–	–	?	±
Goursand, 2009 [24]	Brazil (Portuguese)	+	?	+	+	?	?	?	±
Barbosa, 2009 [25]	Brazil (Portuguese)	+	+	+	+	+	±	?	±
McGrath, 2007 [26]	Hong Kong (Cantonese)	±	?	±	±	+	±	?	±
Pipovic, 2024 [27]	Croatia (Croatian)	+	+	+	+	±	+	?	±
Purohit, 2021 [28]	India (Hindi)	±	–	±	?	+	±	?	±
Vinayagamoorthy, 2020 [29]	India (Kannada)	±	–	±	±	+	±	?	±
Kumar, 2016 [30]	India (Telugu)	±	+	±	+	+	±	?	±
Abanto, 2015 [31]	Peru (Spanish)	+	+	+	+	+	±	?	±

Note: Translation and Cross-Cultural Adaptation followed the Principles of Good Practice by Wild et al. (2005) [14]

“+” = Step clearly described and fully compliant with the Principles of Good Practice

“±” = Step partially performed or reported, with deviation from at least one core recommendation

“–” = Step explicitly not performed or clearly omitted from the reported process

“?” = Step insufficiently described to determine compliance

### Measurement properties of the FIS

Tables 3 and 4 summarise the psychometric properties of the FIS based on updated COSMIN criteria [15]. The average FIS-14 score ranged from 2.7 to 25.2, while the average FIS-8 score ranged from 2.3 to 7.2. Floor and ceiling effects were reported in six studies [5, 23, 24, 27, 28, 31]. Ceiling effects were negligible, but floor effects were more prominent, particularly in the Libyan Arabic [23] (18.7%), Brazilian Portuguese [24] (17.9%), Croatian [27] (23.0%) and Peruvian Spanish [31] (35.5%) versions.

#### Structural validity

Only two studies evaluated structural validity: The original English version [5] identified a three-factor structure (Parental/Family Activity, Parental/Family Emotions and Family Conflict), and the Croatian version [27] reported a four-factor structure, including a one-item Financial Burden factor. Both studies explained > 50% variance, meeting COSMIN standards [15]. Neither conducted confirmatory factor analysis (CFA). No study reported Comparative Fit Index (CFI), Root Mean Square Error of Approximation (RMSEA), and Standardized Root Mean Square Residual (SRMR), representing a major gap.

#### Internal consistency

Internal consistency was evaluated in 13 of the 14 included studies, [5, 19, 21–31] and the exact Cronbach alpha values for the total scale and all subdomains are presented in Supplementary Table S1. Overall alpha coefficients for the FIS-14 ranged from 0.79 to 0.88, whereas the FIS-8 showed a broader range from 0.52 to 0.79, with the Omani Arabic version [21] reporting the lowest value. Subdomain-level findings were more heterogeneous: Parental/Family Activity (α = 0.48–0.87) and Parental Emotions (α = 0.59–0.78) generally performed acceptably, while Family Conflict showed wider variation (α = 0.52–0.83). The Financial Burden domain showed the weakest reliability overall, with values as low as 0.23 [29] and several studies not reporting this domain [5, 19, 20, 24–27]. These results suggest that although total-scale internal consistency was satisfactory, the stability of specific subdomains, particularly Family Conflict and Financial Burden, varied substantially across cultural settings.

#### Test–retest reliability

Test–retest reliability was examined in 12 studies, [5, 19, 22–31] with ICC values, sample sizes and retest intervals summarised in Supplementary Table S2. Retest intervals most commonly ranged from 1 to 3 weeks, with several studies explicitly using a 2-week interval [5, 19, 22, 23, 25, 27, 29, 30]. Overall ICC values for the FIS-14 ranged from 0.80 to 0.96, whereas for the FIS-8 ranged from 0.75 to 0.93, indicating acceptable stability of total FIS scores across repeated measurements. Subdomain ICCs were generally acceptable (≥ 0.70) but showed greater variability: Parental/Family Activity (ICC: 0.76–1.00), Parental Emotions (ICC: 0.69–0.96), and Family Conflict (ICC: 0.32–0.96), with the Croatian version [27] showing the weakest performance. Financial Burden showed the greatest inconsistency, with ICCs spanning 0.00–0.92 [22, 24, 26–28, 31].

#### Measurement error

Only one study (the Croatian version) [27] reported measurement error, with SEM values of 0.7 (FIS-14) and 0.6 (FIS-8) and an SDC of 1.8. No studies reported limits of agreement.

#### Construct validity

Construct validity was evaluated in all included studies through convergent and/or known-groups validity. The findings are summarised in Table 4 and detailed in Supplementary Table S3. While convergent validity was generally supported, known-groups validity showed greater variability across oral conditions and cultural contexts.

##### Convergent validity

Across the 10 studies that reported correlation coefficients, [5, 19, 23–28, 30, 31] convergent validity was generally supported, with correlations between total FIS scores and global ratings of child oral health ranging from *ρ* = 0.11 to 0.31, [5, 19, 24–26, 28, 30, 31] and correlations with overall child well-being ranging from *ρ* = 0.11 to 0.48 [5, 19, 23–28, 30, 31]. These represent weak-to-moderate associations, consistent with the a priori hypotheses that poorer perceived oral health or well-being should correspond to higher family impact. The only exception was the Omani Arabic version, which showed no meaningful correlations with global ratings of oral health [21]. Studies that performed adjusted analyses (e.g., Saudi Arabic [22] and Hindi [28] versions) confirmed that these associations persisted after accounting for covariates.

##### Known-groups validity

Known-groups validity was examined in 13 studies across various clinical conditions, including dental caries, malocclusion, oral hygiene, gingivitis, enamel opacities, and fluorosis [5, 19, 21–31]. Evidence was strongest for malocclusion, with five versions reporting significantly higher FIS scores among children with more severe malocclusion, supporting predefined hypotheses, [25, 27, 29, 30, 31] while one study found no significant difference [19]. For dental caries, findings were more variable: five studies reported higher FIS scores in children with caries, [5, 23, 27, 28, 31] while four studies found no significant differences [19, 25, 29, 30]. For oral hygiene and gingival health, two studies showed clear but non-significant gradients in FIS scores across severity levels, [22, 23] while one study reported significantly higher FIS scores in children with poorer oral hygiene and increased odds of family impact (OR = 2.99, 95% CI: 1.52–5.89) [28]. Enamel opacities showed no significant associations, [19] whereas fluorosis demonstrated a clear difference between severity groups [30]. Overall, known-groups validity of the FIS was well supported for malocclusion and, to a lesser extent, for oral hygiene status, whereas evidence was inconsistent for dental caries and gingival conditions and absent or limited for enamel opacities and fluorosis.

#### Responsiveness

Responsiveness was examined in two studies [20, 27]. The Canadian version [20] reported moderate effect sizes among orthodontic patients (*n* = 26), and the Croatian longitudinal subset [27] demonstrated significant pre–post changes following orthodontic treatment.

#### Cross-cultural validity and criterion validity

Cross-cultural validity and criterion validity were not evaluated in any of the included studies.

**Table 3:** Summary of psychometric properties of the family impact scale (FIS) in the considered studies based on the COSMIN framework

Study (Country, Language)	FIS Total Score (Mean ± Standard Deviation)	Floor/Ceiling Effects	Structural Validity	Internal Consistency (α)	Test–Retest Reliability/ Measurement Error
**FIS-14**					
Locker, 2002 [5] (Canada, English)	8.28 ± 6.83	Floor: 10.20% Ceiling: 0%	EFA (3 factors, 54.7% variance)	α = 0.83	ICC = 0.80 (95% CI: NA)
Marshman, 2007 [19] (United Kingdom, English)	2.70 ± 4.10	Floor: NA Ceiling: NA	NA	α = 0.82	ICC = 0.92–0.95
Agou, 2008 [20] (Canada, English)	Pre-orthodontic: 20.7 (NR) Post-orthodontic: 17.6 (NR)	Floor: NA Ceiling: NA	NA	NA	NA
Quadri, 2021 [22] (Saudi Arabia, Arabic)	NA	Floor: NA Ceiling: NA	NA	α = 0.86	ICC = 0.90 (95% CI: 0.80–0.93)
Goursand, 2009 [24] (Brazil, Portuguese)	NA	Floor: 17.90% Ceiling: 0.80%	NA	α = 0.79	ICC = 0.83 (95% CI: NA)
Barbosa, 2009 [25] (Brazil, Portuguese)	NA	Floor: NA Ceiling: NA	NA	α = 0.87	ICC = 0.90 (95% CI: 0.83–0.93)
McGrath, 2007 [26] (Hong Kong, Cantonese)	5.20 ± 5.50	Floor: NA Ceiling: NA	NA	α = 0.82	ICC = 0.87 (95% CI: 0.83–0.92)
Pipovic, 2024 [27] (Croatia, Croatian)	3.10 ± 4.40	Floor: 23.0% Ceiling: 0%	EFA (4 factors, 56% variance)	α = 0.81	ICC = 0.86 (95% CI: 0.72–0.94) ME = 0.7; SDC = 1.8
Purohit, 2021 [28] (India, Hindi)	25.16 ± 5.96	Floor: 4.0% Ceiling: 0%	NA	α = 0.82	ICC = 0.86 (95% CI: 0.73–0.92)
Vinayagamoorthy, 2020 [29] (India, Kannada)	Caries: 12.63 ± 6.39 No caries: 12.79 ± 7.35 Malocclusion: 15.54 ± 6.53 Normal occlusion: 8.44 ± 4.66	Floor: NA Ceiling: NA	NA	α = 0.88	ICC = 0.95 (95% CI: NA)
Abanto, 2015 [31] (Peru, Spanish)	5.20 ± 5.86	Floor: 35.5% Ceiling: 0%	NA	α = 0.84	ICC = 0.96 (95% CI: 0.90–0.98)
**FIS-8**					
Al-Riyami, 2016 [21] (Oman, Arabic)	2.30 ± 2.20	Floor: NA Ceiling: NA	NA	α = 0.52	NA
Mansur, 2022 [23] (Libya, Arabic)	7.20 ± 6.00	Floor: 18.7% Ceiling: 0%	NA	α = 0.79	ICC = 0.93 (95% CI: 0.85–0.97)
Pipovic, 2024 [27] (Croatia, Croatian)	2.40 ± 3.20	Floor: NA Ceiling: NA	NA	α = 0.73	ICC = 0.87 (95% CI: 0.73–0.94) ME = 0.6; SDC = 1.8
Kumar, 2016 [30] (India, Telugu)	4.44 ± 5.08	Floor: NA Ceiling: 0%	NA	α = 0.78	ICC = 0.75 (95% CI: 0.68–0.81)

NA = not applicable; NR = not reported; EFA = exploratory factor analysis; α = Cronbach’s alpha (internal consistency); ICC = intraclass correlation coefficient; ME = measurement error; SDC = smallest detectable change; *r* = Spearman’s rank correlation coefficient or *Pearson correlation coefficient; all values are presented with two decimal places for consistency

**Table 4:** Construct validity of the family impact scale (FIS) in the included studies (convergent and known-group validity)

Study (Country, Language)	Convergent Validity (Global Rating Correlation)	Condition Evaluated	Known-Groups Validity (Test & Results)
**FIS-14**			
Locker, 2002 [5] (Canada, English)	Oral hygiene: *ρ* = 0.24 Well-being: *ρ* = 0.47	Diagnostic groups (3 groups: dental caries (24.80%), orthodontic (45.30%), orofacial (29.90%))	• Significant differences in FIS score (10/14 items, Kruskal–Wallis test)
		Dental caries (WHO criteria: mean ± SD of decayed teeth = 5.20 ± 3.20, decayed surfaces = 10.20 ± 7.40)	• Significant correlation of FIS score with number of decayed surfaces (*r* = 0.30, test not specified)
		Malocclusion (Class I, class I div 1, class II div II, class III, oligodontia, not known)	• Known-groups comparison not performed due to nominal scale
Marshman, 2007 [19] (United Kingdom, English)	Oral hygiene: *ρ* = 0.21–0.27 Well-being: ρ = 0.41–0.42	Dental caries (WHO criteria: total DMFT = 1.23, decayed teeth = 0.41, missing teeth due to caries = 0.06, filled teeth = 0.77)	• No significant association between FIS score and caries parameters (Spearman’s rank correlation; Mann–Whitney U test)
		Malocclusion (IOTN DHC classification; 68% had malocclusion)	• No significant association between FIS score and IOTN classification (Spearman’s rank correlation; Mann–Whitney U test)
		Teeth opacities (absent vs. present)	• No differences in FIS scores (Mann–Whitney U test)
Quadri, 2021 [22] (Saudi Arabia, Arabic)	NA	Oral hygiene (parents perceived oral hygiene status; 5 groups: very poor, poor, fair, good, very good)	• Clear gradient in mean and median FIS scores by hygiene, but no significant differences (Kruskal–Wallis test). • Better hygiene significantly associated with less family impact (Fisher’s exact test, logistic regression)
Goursand, 2009 [24] (Brazil, Portuguese)	Oral hygiene: *ρ* = 0.25 Well-being: *ρ* = 0.38	Diagnostic groups (2 groups: dental caries vs. orthodontic)	• No differences in mean or median FIS score (test not specified)
Barbosa, 2009 [25] (Brazil, Portuguese)	Oral hygiene: *ρ* = 0.22 Well-being: *ρ* = 0.30	Dental caries (WHO criteria; 3 groups: dmft/DMFT = 0, 1–2, ≥ 3)	• Clear gradient in mean and median FIS scores (total score and all subscales), but no significant differences (Mann–Whitney U test)
		Malocclusion (DAI; 4 groups: none–minor, definitive, severe, handicapping)	• Significant differences in total FIS score and score on Parental Emotions subscale between none–minor and severe/handicapping malocclusion groups
McGrath, 2007 [26] (Hong Kong, Cantonese)	Oral hygiene: *ρ* = 0.31 Well-being: *ρ* = 0.48	Diagnostic groups (2 groups: pedodontic vs. orthodontic)	• Significantly higher FIS and Parental Emotions subscale scores in the orthodontic group (Mann–Whitney U test)
Pipovic, 2024 [27] (Croatia, Croatian)	Well-being: *r* = 0.33	Dental caries (WHO criteria; 2 groups: caries-free vs. caries experience)	• Significantly higher FIS scores (total score and scores on the Parental Activity and Parental Emotions subscales) in the caries experience group (Student’s *t* test)
		Malocclusion (IOTN DHC; 2 groups: IOTN ≤ 2 vs. IOTN ≥ 3)	• Significantly higher FIS scores (total score and scores on the Parental Activity and Parental Emotions subscales) in the IOTN DHC ≥ 3 group (Student’s *t* test)
Purohit, 2021 [28] (India, Hindi)	Oral hygiene: *ρ* = 0.29 Well-being: *ρ* = 0.20	Dental caries (WHO criteria; 3 groups: DMFT = 0, 1–2, 3–5)	• Significant differences in mean FIS score (one-way ANOVA). • DMFT was a significant independent predictor of total FIS after adjusting for oral health rating, SES and covariates (R² = 0.138, linear regression) • Higher DMFT increased odds of family impact (OR = 1.37, 95% CI: 1.08–2.33, *p* < 0.05, logistic regression)
		Oral hygiene status (Debris Index, Calculus Index, Simplified OHI-S; 3 groups: good, fair, poor)	• Significantly higher FIS in the poorer oral hygiene group (one-way ANOVA) • Oral hygiene rating predicted FIS in linear regression. • Poor oral hygiene increased odds of family impact (OR = 2.99, 95% CI: 1.52–5.89, *p* < 0.001, logistic regression)
Vinayagamoorthy, 2020 [29] (India, Kannada)	NA	Dental caries (WHO criteria; 2 groups: caries vs. non-caries; 58.20% with caries)	• No difference in mean FIS score (test not specified)
		Malocclusion (DAI criteria; 2 groups: malocclusion vs. normal occlusion; 59.90% with malocclusion)	• Significantly higher mean FIS scores (total and all subscales) in the malocclusion group (Mann–Whitney U test) • Malocclusion was a significant predictor of higher FIS scores (adjusted IRR = 1.86, 95% CI: 1.60–2.17, *p* < 0.001, negative binomial regression)
Abanto, 2015 [31] (Peru, Spanish)	Oral hygiene: *ρ* = 0.19 Well-being: *ρ* = 0.21	Dental caries (WHO criteria; 2 groups: DMFT = 0 vs. DMFT ≥ 1; 54% had caries)	• Significantly higher median FIS scores (total and all subscales) in the caries group (Mann–Whitney U test)
		Malocclusion (DAI criteria; 2 groups: with malocclusion vs. without malocclusion; 74% had malocclusion)	• Significantly higher median FIS scores (total and all subscales) in the malocclusion group (Mann–Whitney U test)
**FIS-8**			
Al-Riyami, 2016 [21] (Oman, Arabic)	Oral hygiene: *p* > 0.05 (ANOVA, Chi-sqaure)	Dental caries (children suffering from toothache: yes vs. no)	• No difference in mean FIS score (test not specified)
Mansur, 2022 [23] (Libya, Arabic)	Well-being: *ρ* = 0.39	Dental caries (WHO criteria; 2 groups: caries-free vs. with caries)	• Significant differences in FIS score (Mann–Whitney U test)
		Gingivitis (gingival status; 3 groups: no inflammation, mild gingivitis, moderate gingivitis)	• Clear gradient in mean FIS-8 score (less family impact with less inflammation), but not statistically significant (Mann–Whitney U test)
Pipovic, 2024 [27] (Croatia, Croatian)	Well-being: *r* = 0.31	Dental caries (WHO criteria; 2 groups: caries-free vs. caries experience)	• Significantly higher mean FIS-8 score in caries experience group (Student’s *t* test)
		Malocclusion (IOTN DHC; 2 groups: IOTN ≤ 2 vs. IOTN ≥ 3)	• Significantly higher mean FIS-8 score in group with IOTN DHC ≥ 3 (Student’s *t* test)
Kumar, 2016 [30] (India, Telugu)	Oral hygiene: *ρ* = 0.11 Well-being: *ρ* = 0.11	Dental caries (WHO criteria; 3 groups: DMFT = 0, 1–3, > 3)	• No differences in median FIS score (Mann–Whitney U test)
		Malocclusion (orthodontic treatment required; yes vs. no)	• Significant differences in median FIS score (Mann–Whitney U test)
		Fluorosis (Dean’s Index; 2 groups: none–mild vs. moderate–severe)	• Significant differences in median FIS score (Mann–Whitney U test)

NA = not applicable; *ρ* = Spearman’s rank correlation coefficient; *r* = Pearson correlation coefficient; R² = coefficient of determination; OR = odds ratio; IRR = incidence rate ratio; DMFT = decayed, missing and filled teeth; IOTN = Index of Orthodontic Treatment Need; DHC = dental health component; WHO = World Health Organization; DAI = Dental Aesthetic Index; all correlation coefficients were statistically significant (*p* < 0.05)

### Evaluation of psychometric measurement properties according to COSMIN criteria

Table 5 summarizes the evaluation of measurement properties across all included studies according to the COSMIN criteria for good measurement properties (version 2.0) [15]. *Content validity* was rated as sufficient in seven studies, including the original version [5] and six translated versions [25–27, 29−31]. In contrast, five studies were rated as indeterminate, primarily due to insufficient reporting of patient involvement, lack of expert review, or absence of descriptions of cognitive interviewing procedures [19, 21, 22, 24, 28]. *Structural validity* was assessed in only two studies: the original English version, [5] which was rated sufficient, and the Croatian version, [27] which was rated indeterminate because factor-analytic methods were incomplete and confirmatory analyses were not performed. *Internal consistency* was rated as sufficient in 11 of the 12 studies reporting alpha values, with the only insufficient rating occurring in the Omani Arabic short version, [21] where Cronbach’s alpha fell below 0.70. *Test–retest reliability* was reported and rated as sufficient across most translations, [5, 19, 22–31] although a few studies [20, 21] did not report reliability data. *Measurement error* was examined in only two studies, [5, 27] both receiving indeterminate ratings because SEM and SDC values were either partially reported or lacked interpretability thresholds. *Hypothesis testing for construct validity* was conducted in 13 studies, with 12 rated as sufficient based on predefined hypotheses being met for convergent or known-groups validity [5, 19, 22–31]; only the Omani Arabic version was rated as insufficient due to non-significant and directionally inconsistent correlations [21]. *Responsiveness* was assessed using comparator instruments or before–after analyses for two translations and was rated as sufficient [20, 27]. No study evaluated *cross-cultural validity* or *criterion validity*; therefore, these domains were not included in Table 5.

Across measurement properties, the most common reasons for downgrading included small sample sizes, lack of transparency in translation and adaptation procedures, absence of CFA, inadequate reporting of reliability statistics (e.g., missing ICC models or confidence intervals), and insufficient methodological detail in content validity procedures.

**Table 5:** Evaluation of measurement properties according to the COSMIN criteria for good measurement properties

Study	Country (language)	Content Validity	Structural Validity	Internal Consistency	Reliability	Measurement Error	Hypothesis Testing for Construct Validity	Responsiveness
Locker, 2002 [5]	Canada (English)	+	+	+	+	?	+	
Marshman, 2007 [19]	United Kingdom (English)	?		+	+		+	
Agou, 2008 [20]	Canada (English)						?	+
Al-Riyami, 2016 [21]	Oman (Arabic)	?		-			-	
Quadri, 2021 [22]	Saudi Arabia (Arabic)	?		+	+		+	
Mansur, 2022 [23]	Libya (Arabic)			+	+		+	
Goursand, 2009 [24]	Brazil (Portuguese)	?		+	+		+	
Barbosa, 2009 [25]	Brazil (Portuguese)	+		+	+		+	
McGrath, 2007 [26]	Hong Kong (Cantonese)	+		+	+		+	
Pipovic, 2024 [27]	Croatia (Croatian)	+	?	+	+	?	+	+
Purohit, 2021 [28]	India (Hindi)	?		+	+		+	
Vinayagamoorthy, 2020 [29]	India (Kannada)	+		+	+		+	
Kumar, 2016 [30]	India (Telugu)	+		+	+		+	
Abanto, 2015 [31]	Peru (Spanish)	+		+	+		+	

+ = sufficient; - = insufficient; ? = indeterminate; blank = not reported

Note: Columns for *Cross-Cultural Validity* and *Criterion Validity* were omitted to enhance readability, as these properties were not assessed for or not applicable to all included studies

### Risk of bias assessment (COSMIN risk of bias checklist)

Table 6 summarises the methodological quality ratings of the 14 included studies based on the COSMIN Risk of Bias Checklist version 3, [15] with detailed appraisal provided in Supplementary Appendix 4 with blank cells indicate properties that were not assessed. Across domains, content validity and PROM development were the weakest areas, whereas construct validity and responsiveness demonstrated stronger methodological quality.

Only one study, the original instrument developed by Locker et al.’s [5] could be evaluated for *PROM development*, and it was rated doubtful due to several methodological details were insufficiently reported, including the use of skilled interviewers, verbatim transcription, and independent coding. The remaining translation studies and did not report developmental procedures; therefore, this domain was not rated for those versions [19–31]. 

For *content validity*, methodological quality was generally low across studies. Two studies [20, 23] did not report any content validity procedures and were therefore unrated. Twelve studies were evaluated using the COSMIN criteria for relevance, comprehensiveness, and comprehensibility, with patient input and expert input separately. Expert involvement was limited: only three studies included experts in evaluating item relevance, comprehensiveness, or comprehensibility, [5, 24, 31] whereas most studies (*n* = 9) relied solely on patient feedback without expert review [19, 21, 22, 25–30]. Content validity based on expert input was predominantly rated “doubtful” due to limited reporting on researcher involvement or expert qualifications [5, 24]. The Peruvian Spanish version [31] was the only adaptation to achieve an “adequate” rating for expert input, although its patient rating remained “doubtful”. All studies that evaluated patient input received “doubtful” ratings [5, 19, 21, 22, 25–31]. Common reasons for downgrading included absence of structured interview guides, [5, 26, 27, 30, 31] unclear interviewer training [5, 26, 27, 30] or transcription procedures, [5, 26, 27, 30, 31] and lack of independent coding [5, 19, 21, 22, 24–26, 28−30].

*Structural validity* was assessed in only two studies [5, 27]. Both relied on exploratory factor analysis (EFA) and met the COSMIN criterion of at least 50% explained variance, resulting in an “adequate” rating for this domain. However, neither study conducted CFA, and both reported multifactorial structures (three factors in the original version [5] and four in the Croatian version [27]). The absence of confirmatory analyses, insufficient reporting of factor loadings, and lack of invariance testing were the main reasons why no study achieved a “very good” rating for structural validity.

*Internal consistency* was rated as “indeterminate” in all 13 studies according to the COSMIN risk of bias criteria because none established unidimensionality prior to interpreting Cronbach’s alpha values [5, 19, 21–31]. Specifically, neither the FIS-14 nor FIS-8 versions were supported by CFA or adequate evidence of structural validity. As a result, internal consistency could not be judged despite the reporting of alpha coefficients. The primary reasons for downgrading were the absence of CFA, limited reporting of dimensional structure, and reliance on alpha values without verification of scale unidimensionality.

*Reliability* was evaluated in 12 studies, with COSMIN ratings ranging from “very good” to “inadequate”. Four studies achieved a “very good” rating, reflecting appropriate retest intervals, confirmation of patient stability, and complete ICC reporting with confidence intervals [5, 24, 25, 27]. Five studies were rated “adequate” due to acceptable ICC values but minor reporting omissions [19, 23, 29–31]. Two studies were rated “doubtful”, primarily because patient stability was not confirmed or ICCs were incompletely reported, [26, 28] and one was rated “inadequate” due to uninterpretable reliability statistics or insufficiently described retest procedures [22]. Two studies did not report reliability data and were therefore not rated [20, 21]. The most common reasons for downgrading were unclear retest intervals, lack of stability confirmation, unspecified ICC model, and missing confidence intervals.

*Measurement error* was evaluated in only two studies. The Croatian version [27] was rated “very good”, as it reported both the standard error of measurement (SEM) and the smallest detectable change (SDC), allowing interpretability. In contrast, the original development study [5] was rated “inadequate”, because although some variability indices were described, they did not meet COSMIN requirements for quantifying measurement error, and interpretability thresholds were not provided. Overall, the absence of SEM and SDC reporting constituted the primary reason for inadequate or missing ratings in this domain.

*Convergent validity* was assessed in 12 studies and was generally strong. Eleven studies were rated “very good” because they used appropriate comparator measures and confirmed the expected direction and magnitude of correlations [5, 19, 20, 23–28, 30, 31]. One studies received an “adequate” rating due to minor limitations such as incomplete reporting of hypotheses or missing information about the comparator instruments [21]. 

*Known-groups validity* was assessed in 13 studies, of which nine received a “very good” rating for employing clearly defined subgroups and appropriate hypothesis testing [5, 19, 23, 25, 27–31]. Two studies were rated “adequate”: the Chinese version provided sufficiently detailed descriptions of most subgroup characteristics, [26] and the Brazilian Portuguese version used appropriate statistical methods despite limited reporting [24]. Two studies were rated “doubtful,” namely the Omani Arabic version, [21] which provided only minimal subgroup definition by classifying children as having toothache or not, and the Saudi Arabic version, [22] which applied suboptimal methods by reporting only significant subdomain comparisons.

*Responsiveness* was evaluated in only two studies, [20, 27] both of which met their predefined hypotheses using appropriate comparator or pre–post analyses and were therefore rated “very good.” The remaining 12 studies were rated not assessed due to the absence of pre–post data, comparator instruments, or a priori hypotheses. No study evaluated *cross-cultural validity* or *criterion validity*, and these domains were therefore omitted from Table 6.

**Table 6:** Summary of risk of bias assessment among the included studies

Study	Country (language)	PROM Development	Content Validity			Structural Validity	Internal Consistency	Reliability	Measurement Error	Hypothesis Testing for Construct Validity		Responsiveness
			Relevance	Comprehensiveness	Comprehensibility					Convergent	Known-Groups	
Locker, 2022 [5]	Canada (English)	D	D^PE^	D^PE^	D^PE^	A	?	VG	IA	VG	VG	
Marshman, 2007 [19]	United Kingdom (English)		D^P^	D^P^	D^P^		?	A		VG	VG	
Agou, 2008 [20]	Canada (English)									VG		VG^OB^
Al-Riyami, 2016 [21]	Oman (Arabic)		D^P^	D^P^	D^P^		?			A	D	
Quadri, 2021 [22]	Saudi Arabia (Arabic)		D^P^	D^P^	D^P^		?	IA			D	
Mansur, 2022 [23]	Libya (Arabic)						?	A		VG	VG	
Goursand, 2009 [24]	Brazil (Portuguese)		D^E^	D^E^	D^E^		?	VG		VG	A	
Barbosa, 2009 [25]	Brazil (Portuguese)		D^P^	D^P^	D^P^		?	VG		VG	VG	
McGrath, 2007 [26]	Hong Kong (Cantonese)		D^P^	D^P^	D^P^		?	D		VG	A	
Pipovic, 2024 [27]	Croatia (Croatian)		D^P^	D^P^	D^P^	A	?	VG	VG	VG	VG	VG^B^
Purohit, 2021 [28]	India (Hindi)		D^P^	D^P^	D^P^		?	D		VG	VG	
Vinayagamoorthy, 2020 [29]	India (Kannada)		D^P^	D^P^	D^P^		?	A			VG	
Kumar, 2016 [30]	India (Telugu)		D^P^	D^P^	D^P^		?	A		VG	VG	
Abanto, 2015 [31]	Peru (Spanish)		D^P^; A^E^	D^P^; A^E^	D^P^; A^E^		?	A		VG	VG	

Abbreviations: VG = very good; A = adequate; IA = inadequate; D = doubtful; ? = indeterminate; blank = not reported

Superscripts: ᴾ = content validity assessed through patient rating; ᴱ = content validity assessed through expert rating; ᴼ = responsiveness

Assessed using comparison with other instruments; ᴮ = responsiveness assessed through before-and-after intervention analysis

## Discussion

Across diverse cultural contexts, the FIS generally demonstrated acceptable internal consistency (α = 0.79–0.88 for FIS-14; 0.52–0.79 for FIS-8) and satisfactory test–retest reliability (ICC = 0.75–0.93 for FIS-14; 0.80–0.96 for FIS-8). Evidence for construct validity was generally supportive, with most studies confirming predefined hypotheses for both convergent and known-groups validity. Convergent validity was indicated by moderate to strong correlations with related oral health–related quality of life instruments, while known-groups validity was most consistently supported for malocclusion and, to a lesser extent, oral hygiene status. Evidence was more variable for dental caries and gingival conditions and limited or inconsistent for enamel opacities and fluorosis. These findings support the FIS as a useful caregiver-reported measure of family impact. However, evidence for its structural validity, measurement error, responsiveness, and cross-cultural validity remained limited. These gaps restrict conclusions about dimensionality, score stability, and sensitivity to change, all of which are essential for the confident application of the FIS in research and clinical settings.

The methodological rigour of *translation and cultural adaptation* processes varied considerably across studies [21–31]. Although many versions described forward–backward translation and some degree of expert input, key stages recommended by international guidelines, including the use of an adequate number of independent translators, reconciliation, independent expert evaluation, cognitive debriefing with a sufficient and representative number of caregivers, and proofreading, were often insufficiently described or omitted [14, 15, 21–31]. In some adaptations, reconciliation or expert review was minimal or absent, and cognitive debriefing was limited by small or unrepresentative samples [21–24, 26, 28, 29]. Proofreading was not reported in any study, and most did not present the finalised instrument in full. Collectively, these shortcomings raise concerns about semantic, content, and conceptual equivalence, potentially compromising cross-cultural comparability. Greater adherence to established reporting frameworks is therefore essential, and users should critically appraise the methodological quality of each version before application.

*Content validity* was weakened by insufficient reporting and inconsistent methodological approaches. Few studies described the qualifications of the experts involved, the use of structured interview guides, or whether interviewers were adequately trained. Independent coding and triangulation of findings were rarely reported. Only the Peruvian Spanish version demonstrated adequate methodological detail, involving both caregivers and a multidisciplinary panel with clearly described procedures [31]. Without rigorous and transparent methods, the risk of bias and conceptual drift increase, undermining confidence that translated items retain relevance, comprehensibility, and representativeness for local populations. To improve methodological rigour, future adaptation studies should explicitly follow COSMIN recommendations by integrating both patient and expert perspectives, ensuring independent qualitative analysis and thoroughly documenting all steps [15, 32]. 

Evidence for *structural validity* remains limited. Only two studies examined the underlying dimensionality using EFA [5, 27]. The original Canadian study identified a three-factor structure, whereas the Croatian version reported a four-factor structure, including the emergence of a single-item Financial Burden factor [5, 27]. These differences may reflect cultural variation in perception of financial strain or differences in healthcare financing systems. Neither study reported item-level analyses, scree plots, CFA or model fit indices such as CFI or RMSEA, which restricts conclusions regarding structural stability or measurement invariance across cultures. Future validation should therefore employ CFA and modern psychometric approaches (e.g., item response theory or Rasch modelling) to verify factor structure, enhance interpretability, and formally test equivalence across diverse populations [15, 32]. 

*Internal consistency* was generally acceptable for FIS-14, with most domains reaching Cronbach’s alphas ≥ 0.70. However, alpha values varied across subdomains, particularly for Parental/Family Activity, Parental emotions, and Family Conflict, which showed lower or inconsistent values in some adaptations [23–25, 27, 31]. The Financial Burden item frequently showed weak reliability or was not reported [5, 19, 20, 24–27, 31]. Despite these acceptable alpha values, COSMIN criteria rated internal consistency as “indeterminate” because unidimensionality was not established by factor analysis [15]. Moreover, reliance on alpha alone may overestimate reliability when items share similar wording. Future studies should therefore evaluate reliability using multidimensional indices (e.g., McDonald’s omega, ordinal alpha, SEM-based reliability) and report domain-specific estimates.

*Test–retest reliability* was generally acceptable, but methodological reporting was inconsistent. Many studies did not specify the ICC model used, omitted confidence intervals, provided insufficient justification for the retest interval, or failed to clearly establish participant stability between administrations [19, 22, 23, 26, 28–31]. To strengthen evidence of temporal reliability, future studies should explicitly report ICC models, provide confidence intervals, justify retest intervals, and document stability assumptions in accordance with COSMIN standards [15, 32]. 

*Measurement error* was assessed in only two studies, [5, 27] and only one reported both the standard error of measurement and the smallest detectable change [27]. These parameters are essential for determining whether observed score differences reflect meaningful change rather than random measurement variation [32]. 

*Construct validity* was supported in most studies, although effect sizes varied. Convergent validity showed weak-to-moderate associations with global oral health and overall well-being ratings (ρ = 0.11–0.48) [5, 19, 23–28, 30, 31]. Known-groups validity was strongest for malocclusion, with consistently higher FIS scores observed in more severe cases across diverse cultures [25, 27, 29–31]. In contrast, associations with dental caries, gingival health, enamel opacities, and fluorosis were less consistent, possibly reflecting differences in disease salience, parental burden, or socio-educational context [5, 19, 24, 27–31]. 

*Criterion validity* was not assessed in any included studies, refleccting the absence of a true gold standard for measuring family impact. Accordingly, and in line with COSMIN methodology, construct validity currently remains the primary basis for evaluating the FIS [15, 32]. Future research should strengthen construct validity by using predefine hypotheses, consistent comparator instruments, and rigorously defined subgroup criteria, particularly in cross-cultural contexts.

*Responsiveness* was assessed in only two studies [20, 27]. The Canadian study reported a moderate effects following orthodontic treatment but was limited by a small sample size, lack of a comparison group, and absence of predefined hypotheses [20]. The Croatian study demonstrated significant pre–post differences but did not meet the COSMIN threshold of ≥ 75% hypothesis confirmation [15, 27]. Although several excluded studies used the FIS before and after interventions (e.g., general anaesthesia, zirconia crowns, routine care), they did not formally assess responsiveness [8, 33–35]. Given the increasing application of the FIS in intervention research, rigorously designed longitudinal studies with hypothesis-driven frameworks and appropriate comparators are needed. Establishing responsiveness is essential for positioning FIS as a clinically meaningful instrument capable of capturing changes following dental interventions [15, 32]. 

*Floor effects* varied widely across studies, ranging from negligible to substantial, whereas *ceiling effects* were consistently minimal [5, 23, 24, 27, 28, 30, 31]. Pronounced floor effects limit sensitivity in low-burden populations and reduce the ability of the FIS to detect meaningful change over time. Importantly, several studies did not report floor and ceiling effects at all, and most restricted reporting to total scores [19–22, 25−27, 29]. Domain-level reporting is essential for understanding the effective measurement range of individual subscales and for informing whether item modification or recalibration is required.

None of the included studies performed *cross-cultural validity* testing (e.g., differential item functioning, multigroup CFA). This represents a major limitation, as translated versions may differ systematically even if they share linguistic equivalence. For example, the UK English version was adapted without an evaluation of equivalence across English-speaking populations,^19^ and the Libyan version adapted from the Oman version without any additional cross-cultural testing [21, 23]. Without formal invariance testing, it is difficult to determine whether observed score differences between populations reflect true differences in family impact or measurement artefacts.

This review’s strengths include the application of two complementary frameworks: the COSMIN and Wild et al., which enabled a comprehensive evaluation of methodological quality and transparency [14, 15]. These frameworks helped systematically identify deficiencies in content validity, cross-cultural adaptation, and reporting. The review also highlights practical implications for clinicians and researchers: FIS-14 is suitable for assessing the family burden of oral diseases, particularly malocclusion, but should be used with caution in cross-cultural comparisons and longitudinal monitoring until gaps in structural validity, responsiveness, and measurement error are addressed. The review further underscores the importance of rigorous translation processes in academic and clinical training, as inconsistencies or omissions in adaptation may compromise measurement accuracy in both practice and education settings.

Several limitations should be acknowledged. First, heterogeneity in design, sample characteristics, and psychometric procedures prevented a meta-analysis, limiting quantitative synthesis. Second, publication bias cannot be excluded, as studies reporting poor measurement properties may be underrepresented. Finally, many included studies predate the international guidelines, which may partially explain methodological gaps such as insufficient content validity or unclear translation procedures [5, 14, 15, 19–26]. 

Despite these limitations, this review provides the most comprehensive synthesis of FIS adaptations to date, offering a structured appraisal of translation processes and psychometric evidence across cultures. By identifying robust findings and recurrent methodological weaknesses, this review serves as a reference point for improving the development, adaptation, and evaluation of family-focused oral health instruments. Strengthening future validation studies through rigorous qualitative procedures, modern psychometric methods, and cross-cultural equivalence testing will enhance the global comparability and clinical utility of the FIS.

## Conclusion

In summary, the evidence from this systematic review indicates that the FIS 14 demonstrates acceptable reliability and construct validity across diverse cultural settings, supporting its use in assessing the family impact of child oral health conditions. However, important psychometric properties, including structural validity, measurement error, responsiveness, and cross-cultural equivalence, remain insufficiently examined. The review underscores the need for standardised translation procedures, clear and comprehensive methodological reporting, and multicentre longitudinal studies to strengthen the measurement robustness of the scale. International collaboration will be essential for establishing the cross-cultural validity of the FIS and improving its global comparability. With continued methodological refinement, the FIS 14 holds strong scientific and practical value for advancing research and enhancing understanding of the family burden associated with child oral health conditions.

## Supplementary Information

Below is the link to the electronic supplementary material.

Supplementary Material 1

Supplementary Material 2

Supplementary Material 3

Supplementary Material 4

Supplementary Material 5

Supplementary Material 6

Supplementary Material 7

## Data Availability

The datasets generated and/or analysed during the current study are available from the corresponding author upon reasonable request.

## Abbreviations

Abbreviation: Full Term
FIS: Family Impact Scale
OHRQoL: Oral Health-Related Quality of Life
COHQOL: Child Oral Health Quality of Life Instrument
P-CPQ: Parental-Caregiver Perceptions Questionnaire
CPQ: Child Perceptions Questionnaire
COSMIN: COnsensus-Based Standards for the Selection of Health Measurement INstruments
PRISMA: Preferred Reporting Items for Systematic Reviews and Meta-Analyses
PROSPERO: International Prospective Register of Systematic Reviews
ICC: Intraclass Correlation Coefficient
EFA: Exploratory Factor Analysis
CFA: Confirmatory Factor Analysis
CFI: Comparative Fit Index
RMSEA: Root Mean Square Error of Approximation
SRMR: Standardized Root Mean Square Residual
IRT: Item Response Theory
DAI: Dental Aesthetic Index
IOTN: Index of Orthodontic Treatment Need
DHC: Dental Health Component
DMFT/dmft: Decayed, Missing and Filled Teeth (permanent/primary)
WHO: World Health Organization
SEM: Standard Error of Measurement
SDC: Smallest Detectable Change
SD: Standard Deviation
CI: Confidence Interval
r: Pearson Correlation Coefficient
ρ: Spearman’s Rank Correlation Coefficient
R²: Coefficient of Determination
OR: Odds Ratio
IRR: Incidence Rate Ratio
ME: Measurement Error
