# Supplementary material for: Translation, cross-cultural adaptation, and psychometric properties of the family impact scale: a COSMIN-based systematic review
Source: Health Qual Life Outcomes. 2025 Dec 30;24:17. doi: 10.1186/s12955-025-02473-w (PMC12859982; doi:10.1186/s12955-025-02473-w)
Supplement: Supplementary file 1 — Supplementary Material 1 [file 12955_2025_2473_MOESM1_ESM.pdf]

Supplementary Appendix 1: Full Search Strategies
PubMed
Search Date: March 31, 2025

((("family impact scale*"[Text Word] OR "fis *"[Text Word] OR "family impact questionnaire*"[Text Word]) AND ("cross-cultural adaptation"[Text Word] OR "translation"[Text Word] OR "validation"[Text Word] OR "adaptation"[Text Word] OR "cultural adaptation"[Text Word] OR "psychometric properties"[Text Word] OR "reliability"[Text Word] OR "validity"[Text Word] OR "internal consistency"[Text Word] OR "test-retest reliability"[Text Word] OR "construct validity"[Text Word] OR "content validity"[Text Word] OR "factor analysis"[Text Word] OR "sensitivity and specificity"[Text Word] OR ("translations"[All Fields] OR "culture"[All Fields] OR "adaptation psychological"[All Fields] OR "Cross-cultural comparison"[All Fields] OR "psychometrics"[All Fields] OR "psychometric instrumentation"[All Fields] OR "reproducibility of results"[MeSH Terms]))) NOT ("Fatigue impact scale"[All Fields] OR (("fear"[MeSH Terms] OR "fear"[All Fields] OR "fear of"[All Fields]) AND ("intimacies"[All Fields] OR "intimacy"[All Fields]) AND ("scale s"[All Fields] OR "scaled"[All Fields] OR "scaling"[All Fields] OR "scalings"[All Fields] OR "weights and measures"[MeSH Terms] OR ("weights"[All Fields] AND "measures"[All Fields]) OR "weights and measures"[All Fields] OR "scale"[All Fields] OR "scales"[All Fields])) OR "fissure integrity"[All Fields] OR "functional independence scale"[All Fields] OR "furcation involvement"[All Fields] OR "fertility intention scale"[All Fields] OR "facilitative interpersonal skills"[All Fields] OR "diabetes family impact scale"[All Fields] OR (("covid 19"[All Fields] OR "covid19"[All Fields] OR "covid 19"[MeSH Terms] OR "covid 19 vaccines"[All Fields] OR "covid 19 vaccines"[MeSH Terms] OR "covid 19 serotherapy"[All Fields] OR "covid 19 serotherapy"[MeSH Terms] OR "covid 19 nucleic acid testing"[All Fields] OR "covid 19 nucleic acid testing"[MeSH Terms] OR "covid 19 serological testing"[All Fields] OR "covid 19 serological testing"[MeSH Terms] OR "covid 19 testing"[All Fields] OR "covid 19 testing"[MeSH Terms] OR "sars cov 2"[All Fields] OR "sarscov2"[All Fields] OR "sarscov 2"[All Fields] OR "sars cov2"[All Fields] OR "sars cov 2"[MeSH Terms] OR "severe acute respiratory syndrome coronavirus 2"[All Fields] OR "2019 ncov"[All Fields] OR (("coronavirus"[MeSH Terms] OR "coronavirus"[All Fields] OR "cov"[All Fields] OR "ncov"[All Fields]) AND 2019/11/01:3000/12/31[Date - Publication])) AND ("familialities"[All Fields] OR "familiality"[All Fields] OR "familially"[All Fields] OR "familials"[All Fields] OR "familie"[All Fields] OR "family"[MeSH Terms] OR "family"[All Fields] OR "familial"[All Fields] OR "families"[All Fields] OR "family s"[All Fields] OR "familys"[All Fields]) AND ("impact"[All Fields] OR "impactful"[All Fields] OR "impacting"[All Fields] OR "impacts"[All Fields] OR "tooth, impacted"[MeSH Terms] OR ("tooth"[All Fields] AND "impacted"[All Fields]) OR "impacted tooth"[All Fields] OR "impacted"[All Fields]) AND ("scale s"[All Fields] OR "scaled"[All Fields] OR "scaling"[All Fields] OR "scalings"[All Fields] OR "weights and measures"[MeSH Terms] OR ("weights"[All Fields] AND "measures"[All Fields]) OR "weights and measures"[All Fields] OR "scale"[All Fields] OR "scales"[All Fields])) OR "femininity ideology scale"[All Fields] OR "dermatitis family impact questionnaire"[All Fields])) AND 2001/01/01:3000/12/31[Date - Publication]

Embase
Search Date: March 31, 2025

('validation'/exp OR 'validation' OR 'adaptation'/exp OR 'adaptation' OR 'cultural adaptation'/exp OR 'cultural adaptation' OR 'psychometric properties'/exp OR 'psychometric properties' OR 'reliability'/exp OR 'reliability' OR 'validity'/exp OR 'validity' OR 'internal consistency'/exp OR 'internal consistency' OR 'test retest reliability'/exp OR 'test retest reliability' OR 'construct validity'/exp OR 'construct validity' OR 'content validity'/exp OR 'content validity' OR 'factor analysis'/exp OR 'factor analysis' OR 'sensitivity and specificity'/exp OR 'sensitivity and specificity' OR 'psychometry'/exp OR 'psychometry' OR 'cultural factor'/exp OR 'cultural factor' OR 'translation'/exp OR 'translation' OR 'cross cultural adaptation'/exp OR 'cross cultural adaptation' OR 'reproducibility'/exp OR 'reproducibility') AND ('family impact scale*' OR 'fis' OR 'family impact questionnaire') NOT ('family intention scale' OR 'fatigue impact scale' OR 'fear of intimacy scale' OR 'fissure integrity' OR 'functional independence scale' OR 'furcation involvement' OR 'fertility intention scale' OR 'facilitative interpersonal skills' OR 'diabetes family impact scale' OR 'ovid-19 family impact scale' OR 'femininity ideology scale' OR 'dermatitis family impact questionnaire') AND [2002-2025]/py NOT 'family intergenerational stress' NOT 'fragility index' NOT 'covid-19 family impact scale' NOT 'image* scale*' NOT 'fuzzy inference system*'

Scopus
Search Date: March 31, 2025

(((((TITLE-ABS ("family impact scale*") OR TITLE-ABS ("FIS-*") OR TITLE-ABS ("family impact questionnaire*"))) AND (((TITLE-ABS ("cross-cultural adaptation")) OR (TITLE-ABS ("translation")) OR (TITLE-ABS ("validation")) OR (TITLE-ABS ("adaptation")) OR (TITLE-ABS ("cultural adaptation")) OR (TITLE-ABS ("reliability")) OR (TITLE-ABS ("psychometric properties")) OR (TITLE-ABS ("validity")) OR (TITLE-ABS ("internal consistency")) OR (TITLE-ABS ("test-retest reliability")) OR (TITLE-ABS ("construct validity")) OR (TITLE-ABS ("content validity")) OR (TITLE-ABS ("factor analysis")) OR (TITLE-ABS ("sensitivity and specificity")) OR (TITLE-ABS ("psychometry")) OR (TITLE-ABS ("reproducibility"))))) AND NOT ((TITLE-ABS ("fuzzy inference system")) OR (TITLE-ABS ("family intention scale")) OR (TITLE-ABS ("fatigue impact scale*")) OR (TITLE-ABS ("fear of intimacy scale*")) OR (TITLE-ABS ("fissure integrity")) OR (TITLE-ABS ("functional independence scale*")) OR (TITLE-ABS ("furcation involvement")) OR (TITLE-ABS ("fertility intention scale")) OR (TITLE-ABS ("facilitative interpersonal skills")) OR (TITLE-ABS ("diabetes family impact scale")) OR (TITLE-ABS ("diabetes family impact scale*")) OR (TITLE-ABS ("COVID-19 family impact scale*")) OR (TITLE-ABS ("femininity ideology scale")) OR (TITLE-ABS ("dermatitis family impact questionnaire*")) OR (TITLE-ABS ("Family Intergenerational Stress")) OR (TITLE-ABS ("fragility ind*")) OR (TITLE-ABS ("fragility index")) OR (TITLE-ABS ("fragility indices")) OR (TITLE-ABS ("image* scale*"))))) AND PUBYEAR > 2001
