# Supplementary material for: Translation, cross-cultural adaptation, and psychometric properties of the family impact scale: a COSMIN-based systematic review
Source: Health Qual Life Outcomes. 2025 Dec 30;24:17. doi: 10.1186/s12955-025-02473-w (PMC12859982; doi:10.1186/s12955-025-02473-w)
Supplement: Supplementary file 2 — Supplementary Material 2 [file 12955_2025_2473_MOESM2_ESM.pdf]

Supplementary Appendix 2: Rubric for Evaluating Translation and Cross-cultural Adaptation based on Wild D,2005

This rubric was developed based on the Principles of Good Practice for the Translation and Cultural Adaptation Process for Patient-Reported Outcomes Measures by Wild et al. (2005). It was used to evaluate whether included studies adhered to the recommended translation and cross-cultural adaptation procedures. Each translation step is rated on a 4-point scale:

fully adherent (+): clearly described and aligned with principles of good practice
partially adherent (±): step implemented but lacks some critical components or clarity
not adherent (–): step may have been conducted, but insufficient information is available to assess
unclear (?): step clearly omitted, not aligned with guidelines, or conducted improperly

Scoring was determined based on the following decision logic:

1. If the step was not described → Score: ?
2. If the step was described →
    a. All components met → Score: +
    b. Some components met → Score: ±
    c. Contradicts guidance or done inadequately → Score: −

**Process steps**	+ **(fully adherent)**	± **(partially adherent)**	− **(not adherent)**	? **(unclear)**
**Forward translation**	At least 2 forward translators translate independently; translators fluent in both the original and target languages; preferably, native speaker of the target language	Only one forward translator; Unclear language proficiency	Described in contradiction to principles or clearly only one poor-quality translator used	Not described or reported
**Reconciliation**	Reconcile the translated versions into a single forward translation by a panel or independent native speaker; panel should be described with specified roles in the study (i.e. panel of key in-country person, forward translators, and project manager	Process described, but roles of involved individuals were unclear or not fully specified	Process described but done incorrectly or without proper comparison	Not described or reported
**Back Translation**	At least one backward translator who is native	Translator qualifications unclear, or	Back translation described improperly or	Not described or reported

Process steps	+ (fully adherent)	± (partially adherent)	− (not adherent)	? (unclear)
	speaker of the original language and fluent in target language	process vaguely described	skipped with poor substitute	
**Back Translation Review and Harmonization**	Original version compared; discrepancies resolved in a structured review by the project team (i.e. project manager, key in-country consultant, and forward/ backward translators)	Review conducted but persons or process not detailed	Done without structured review or in contradiction to guidelines	Not described or reported
**Cognitive Debriefing**	5-8 native speakers representing target population; process well-documented	Fewer than 5 or unclear demographics	Not representative participants or methodology clearly flawed	Not described or reported
**Review of Cognitive Debriefing Results and Finalization**	Clarify the topics being assessed; describe the adjustments made if the modification is needed; decision made by at least two people (i.e. project manager and key in-country person)	Review occurred but unclear who reviewed or what changed	Only one person involved or no evidence of review	Not described or reported
**Proofreading**	Final version reviewed and corrected for minor errors such as typographic or grammatical issues	Proofreading mentioned, but no details on outcomes	Proofreading described vaguely or results not addressed	Not described or reported
**Final reported**	Final report includes complete documentation of all steps and the final translated version (or clear access to it)	Final report describes the process adequately, but does not show or link to the final translated version	Final version not shown, and the description of the process is superficial, missing critical steps or rationale	Not described or reported
