# Supplementary material for: Translation, cross-cultural adaptation, and psychometric properties of the family impact scale: a COSMIN-based systematic review
Source: Health Qual Life Outcomes. 2025 Dec 30;24:17. doi: 10.1186/s12955-025-02473-w (PMC12859982; doi:10.1186/s12955-025-02473-w)
Supplement: Supplementary file 3 — Supplementary Material 3 [file 12955_2025_2473_MOESM3_ESM.pdf]

Supplementary Appendix 3: List of Excluded Studies with Reasons

The table below provides a list of full-text articles that were excluded after full-text review, along with the specific reasons and additional notes for exclusion.

Author (Year), Title	Reasons for exclusion	Note
Malden et al. (2008), Changes in parent-assessed oral health-related quality of life among young children following dental treatment under general anaesthetic	Population outside target age range	study assessed oral health–related quality of life in young children under 6 years of age (mean age: 70.2 months) following general anaesthesia.
Thomson et al. (2011), Assessing change in the family impact of caries in young children after treatment under general ana	Population outside target age range	study focused on young children under 6 years (mean age: 70.2 months) receiving dental treatment under general anaesthesia
Thomson et al. (2013), Short-form versions of the Parental-Caregivers Perceptions Questionnaire and the Family Impact Scale	Population outside target age range	study involved development and testing of short forms (including FIS) in preschool-aged population (mean age in study groups: 4.8 and 5.5 years)
Thomson et al. (2014), Comparison of the ECOHIS and short-form P-CPQ and FIS scales	Population outside target age range	study compared instruments (FIS, P-CPQ, ECOHIS) in children under 6 years (mean age in study groups: 4.8 and 5.5 years)
Thomson et al. (2022), Concurrent validity of the short-form Family Impact Scale (FIS-8) in 4-year-old US children	Population outside target age range	study evaluated FIS-8 in 4-year-old children
Baghdadi et al. (2014), Effects of Dental Rehabilitation under General Anesthesia on Children's Oral-Health-Related Quality of Life: Saudi Arabian Parents' Perspectives	Population outside target age range	study focused on young children (mean age 6.3 years) undergoing dental rehabilitation
Baghdadi et al. (2014), Effects of dental rehabilitation under general anesthesia on children's oral health-related quality of life using proxy short versions of OHRQoL instruments	Population outside target age range	Study conducted for children at young age; mean age near lower cutoff (6.07–6.24 years); study population not clearly within 6–14 years target range
Khoun et al. (2018), Oral health-related quality of life in young Cambodian children: a validation study with a focus on	Population outside target age range	Study population primarily included children with cleft lip/palate under 6 years (age range 2–8 years); does not align with inclusion criteria of 6–14 years for FIS validation

Author (Year), Title	Reasons for exclusion	Note
children with cleft lip and/or palate		
Keränen et al. (2021), Validating a short form of the Parental-Caregivers Perceptions Questionnaire (P-CPQ) and the Family Impact Scale (FIS) in Finnish language	Population outside target age range	study validated short-form FIS in Finnish in preschool-aged children (mean age: 5 years)
Cartes-Velásquez et al. (2023), Validation of family impact scale to assess oral health-related quality of life in parents of chilean preschoolers	Population outside target age range	study validated the FIS in parents of preschoolers (mean age: 5.69 years)
Hasanzadeh et al. (2014), Coping strategies and psychological distress among mothers of patients with nonsyndromic cleft lip and palate and the family impact of this disorder	Outcome: FIS used but measurement properties not evaluated	FIS used to assess family impact, but no psychometric evaluation of the instrument was conducted
Fernandes et al. (2016), The impact of the oral condition of children with sickle cell disease on family quality of life	Outcome: FIS used but measurement properties not evaluated	FIS used in families of children with sickle cell disease; study did not assess reliability, validity, or other measurement properties
Bani et al. (2017), How Does Dental Trauma Affect the Quality of Life in Turkish Families?	Outcome: FIS used but measurement properties not evaluated	Study examined dental trauma and its impact on families using FIS, but did not evaluate the instrument's measurement properties
Aurlene et al. (2022), The Role of Parenting Practices on the Parent Perceived Impact of Child Oral Health on Family Wellbeing	Outcome: FIS used but measurement properties not evaluated	Used FIS to measure parent-perceived impact but did not assess its psychometric performance
Santiago et al. (2022), A network psychometric validation of the Children Oral Health-Related Quality of Life (COHQoL) questionnaire among Aboriginal and/or Torres Strait Islander children	Outcome: FIS used but measurement properties not evaluated	Study focused on validation of the COHQoL; although FIS may have been mentioned or used, it was not the instrument under validation

Author (Year), Title	Reasons for exclusion	Note
Arrow et al. (2021), Evaluation of the ECOHIS and the CARIES-QC among an Australian "Aboriginal" population	Outcome: Did not assess the Family Impact Scale	FIS referred to ECOHIS subscale, not the standalone Family Impact Scale (FIS); measurement properties of FIS were not evaluated
Umay et al. (2019), Reliability and validity of the pediatric feeding and swallowing disorders family impact scale for Turkish children with cerebral palsy by endoscopic ev aluation	Outcome: Did not assess the Family Impact Scale	Different instrument; study validated a condition-specific family impact scale for feeding and swallowing disorders, not the Family Impact Scale (FIS) by Jokovic et al.
Aguiar et al. (2024), Validity and reliability of the Brazilian version of the Illness Perception Questionnaire-Revised for Dental	Outcome: Did not assess the Family Impact Scale	Study focused on Illness Perception Questionnaire-Revised for Dental (IPQ-RD); short form of the Family Impact Scale (FIS-4) was used as secondary measure, not the focus of validation; psychometric properties of the FIS were not assessed.
Zaror et al. (2019), Assessing oral health-related quality of life in children and adolescents: a systematic review and standardized comparison of available instruments	Ineligible publication type	systematic review, not an original validation study of the FIS
Campbell et al. (2016), Review: Psychometric properties of the Arabic version of the PedsQL™ Family Impact Scale	Ineligible publication type	Review narrative review, not a primary study of the FIS
