# Supplementary material for: Translation, cross-cultural adaptation, and psychometric properties of the family impact scale: a COSMIN-based systematic review
Source: Health Qual Life Outcomes. 2025 Dec 30;24:17. doi: 10.1186/s12955-025-02473-w (PMC12859982; doi:10.1186/s12955-025-02473-w)
Supplement: Supplementary file 5 — Supplementary Material 5 [file 12955_2025_2473_MOESM5_ESM.pdf]

**Supplementary Table S1. Internal consistency of the Family Impact Scale reported as Cronbach's alpha for overall and subdomain scores.**

| Study | Country (Language) | Overall | Parental/ Family Activity | Parental Emotions | Family Conflict | Financial Burden |
|---|---|---|---|---|---|---|
| **FIS-14** | | | | | | |
| Locker, 2002[5] | Canada (English) | 0.83 | 0.72 | 0.70 | 0.64 | NR |
| Marshman, 2007[19] | United Kingdom (English) | 0.82 | NR | NR | NR | NR |
| Agou, 2008[20] | Canada (English) | NR | NR | NR | NR | NR |
| Quadri, 2021[22] | Saudi Arabia (Arabic) | 0.86 | 0.87 | 0.77 | 0.80 | 0.90 |
| Goursand, 2009[24] | Brazil (Portuguese) | 0.79 | 0.59 | 0.62 | 0.52 | NR |
| Barbosa, 2009[25] | Brazil (Portuguese) | 0.87 | 0.77 | 0.59 | 0.77 | NR |
| McGrath, 2007[26] | Hong Kong (Cantonese) | 0.82 | 0.73 | 0.68 | 0.74 | NR |
| Pipovic, 2024[27] | Croatia (Croatian) | 0.81 | 0.69 | 0.61 | 0.60 | NR |
| Purohit, 2021[28] | India (Hindi) | 0.82 | 0.81 | 0.78 | 0.83 | 0.77 |
| Vinayagamoorthy, 2020[29] | India (Kannada) | 0.88 | 0.48 | 0.78 | 0.76 | 0.80 |
| Abanto, 2015[31] | Peru (Spanish) | 0.84 | 0.77 | 0.70 | 0.68 | 0.23 |
| **FIS-8** | | | | | | |
| Al-Riyami, 2016[21] | Oman (Arabic) | 0.52 | NR | NR | NR | – |
| Mansur, 2022[23] | Libya (Arabic) | 0.79 | 0.60 | 0.61 | 0.67 | – |
| Pipovic, 2024[27] | Croatia (Croatian) | 0.73 | NR | NR | NR | – |
| Kumar, 2016[30] | India (Telugu) | 0.78 | NR | NR | NR | – |

NR = Not Reported; – = Not applicable (domain not included in the FIS-8).
