# Supplementary material for: Translation, cross-cultural adaptation, and psychometric properties of the family impact scale: a COSMIN-based systematic review
Source: Health Qual Life Outcomes. 2025 Dec 30;24:17. doi: 10.1186/s12955-025-02473-w (PMC12859982; doi:10.1186/s12955-025-02473-w)
Supplement: Supplementary file 6 — Supplementary Material 6 [file 12955_2025_2473_MOESM6_ESM.pdf]

Supplementary Table S2. Test–retest reliability of the Family Impact Scale reported as intraclass correlation coefficients (ICC) with 95% confidence intervals for overall and subdomain scores.

Study	Country (Language)	No., time interval	Overall	Parental/ Family Activity	Parental Emotions	Family Conflict	Financial Burden
**FIS-14**							
Locker, 2002[5]	Canada (English)	56, 2 wk	0.80 (NR)	NR	NR	NR	NR
Marshman, 2007[19]	United Kingdom (English)	NR, 2 wk	0.92–0.95	NR	NR	NR	NR
Agou, 2008[20]	Canada (English)	NR	NR	NR	NR	NR	NR
Quadri, 2021[22]	Saudi Arabia (Arabic)	40, 2 wk	0.90 (0.80–0.93)	0.92 (0.68–0.96)	0.76 (0.58–0.84)	0.82 (0.58–0.90)	0.92 (0.68–0.94)
Goursand, 2009[24]	Brazil (Portuguese)	53, 3 wk	0.83 (0.71-0.90)	0.84 (0.72-0.91)	0.69 (0.46-0.82)	0.74 (0.54-0.85)	0.59 (0.29-0.76)
Barbosa, 2009[25]	Brazil (Portuguese)	20, 2 wk	0.90 (0.83–0.93)	0.76 (0.58–0.84)	0.69 (0.51–0.82)	0.86 (0.70–0.89)	NR
McGrath, 2007[26]	Hong Kong (Cantonese)	54, 3 wk	0.87 (0.83–0.92)	0.76 (0.66–0.84)	0.71 (0.58–0.80)	0.75 (0.60–0.84)	0.82 (0.72–0.88)
Pipovic, 2024[27]	Croatia (Croatian)	26, 2 wk	0.86 (0.72–0.94)	1.00 (1.00–1.00)	0.81 (0.62–0.91)	0.32 (–0.07–0.62)	0.00 (–0.38–0.38)
Purohit, 2021[28]	India (Hindi)	NR	0.86 (0.73–0.92)	0.84 (0.73–0.9)	0.80 (0.71–0.92)	0.83 (0.71–0.93)	0.81 (0.79–0.89)
Vinayagamoorthy, 2020[29]	India (Kannada)	25, 2 wk	0.95 (NR)	0.95 (NR)	0.96 (NR)	0.91 (NR)	0.51 (NR) (kappa)
Abanto, 2015[31]	Peru (Spanish)	200, 1–2 wk	0.96 (0.90–0.98)	0.97 (0.94–0.94)	0.96 (0.93–0.98)	0.96 (0.91–0.98)	0.78 (0.67–0.85)
**FIS-8**							
Al-Riyami, 2016[21]	Oman (Arabic)	NR	NR	NR	NR	NR	–
Mansur, 2022[23]	Libya (Arabic)	25, 2 wk	0.93 (0.85–0.97)	0.88 (0.74–0.94)	0.86 (0.71–0.94)	0.64 (0.33–0.82)	–
Pipovic, 2024[27]	Croatia (Croatian)	25, 2 wk	0.87 (0.73–0.94)	NR	NR	NR	–
Kumar, 2016[30]	India (Telugu)	161, 2 wk	0.75 (0.68–0.81)	NR	NR	NR	–

No = number of participants; NR = Not Reported; – = Not applicable (domain not included in the FIS-8).
