# Supplementary material for: Translation, cross-cultural adaptation, and psychometric properties of the family impact scale: a COSMIN-based systematic review
Source: Health Qual Life Outcomes. 2025 Dec 30;24:17. doi: 10.1186/s12955-025-02473-w (PMC12859982; doi:10.1186/s12955-025-02473-w)
Supplement: Supplementary file 7 — Supplementary Material 7 [file 12955_2025_2473_MOESM7_ESM.pdf]

Supplemental Table S3: Detail of known-groups validity of the family impact scale in the included studies

Study	Condition Evaluated	Hypothesis testing (e.g. group Comparison)	Main Findings (Statistical Test Used)
Locker, 2022[5]	Diagnostic groups (3 groups)	Diagnosis: 3 gr.:<br>gr I: Dental caries (24.8%)<br>gr II: Orthodontic (45.3%)<br>gr III: Orofacial (29.9%)	Significant differences were found in 10 of 14 FIS items across the three groups, with the highest mean scores in certain items:<br>- pediatric gr.: time off, attention, upset, future concerns, guilt, and jealousy<br>- orthodontic gr.: interrupt, sleep, conflict<br>- orofacial gr.: financial difficulties<br>(Kruskal-Wallis's test)
	Dental caries	Number of decayed teeth (mean ± SD = 5.2 ± 3.2) Number of decayed surfaces<br>(mean ± SD = 10.2 ± 7.4)	FIS correlated with decayed surfaces ($r = 0.30$, $p < .05$, not specified type of statistics used)
	Malocclusion	Type of malocclusion	not performed due to nominal scale
Marshman, 2007[19]	Dental caries	Total DMFT (mean = 1.23)<br>Decayed teeth (mean = 0.41)<br>Missing teeth due to caries (mean = 0.06)<br>Filled teeth (mean = 0.77)	No significant association between FIS and studied caries parameters found regardless of "don't know" response handling. (Spearman rank correlation, Mann–Whitney U test)
	Malocclusion	IOTN DHC classification (68% had malocclusion)	No significant association between FIS and IOTN found regardless of "don't know" response handling. (Spearman rank correlation, Mann–Whitney U test)
	Teeth Opacities	Teeth opacities: 2 gr:<br>gr I: Absent<br>gr II: Present	No significant difference in mean FIS scores between groups. ($p > .05$, Mann–Whitney U test)
Agou, 2008[20]	Malocclusion	-	No group comparisons in this study.

Study	Condition Evaluated	Hypothesis testing (e.g. group Comparison)	Main Findings (Statistical Test Used)
Al-Riyami, 2016[21]	Dental caries	Children suffering toothache: yes vs. no	No significant difference in mean FIS score between the 2 groups (type of statistics not clearly specified).
Quadri, 2021[22]	Oral hygiene	Parents perceived oral hygiene status of their children: 5 gr.:<br>gr I: very poor<br>gr II: poor<br>gr III: fair<br>gr IV: good<br>gr V: very good	Clear, but not statistically significant, gradient of mean and median of FIS across severity of oral health status in overall, 4 subscales and each item ($p > .05$, Kruskal–Wallis test).
			Better oral hygiene was associated with a lower frequency of family impact ($p < .001$, Fisher exact test)
			Lower frequency of family impact corresponds to better oral health status of children ($p < .001$, binary logistic regression)
Mansur, 2022[23]	Dental caries	WHO criteria: 2 gr:<br>gr I: caries free<br>gr II: with caries	Significant differences of FIS between the two groups (Mann–Whitney U test).
	Gingivitis	Gingival status:<br>gr I: no inflammation,<br>gr II: mild gingivitis<br>gr III: moderate gingivitis	Clear, but not statistically significant, gradient (Less family impact in less gingival inflammation groups) was observed in mean FIS-8 scores across the groups ($p > .05$, Mann–Whitney U test).
Goursand, 2009[24]	Diagnostic groups (2 groups)	Treatment required: 2 gr.<br>gr I: Dental caries<br>gr II: Orthodontic	No statistically significant difference in mean and median of overall FIS and in the 4 subscales between the two groups (type of statistics not clearly specified).

Study	Condition Evaluated	Hypothesis testing (e.g. group Comparison)	Main Findings (Statistical Test Used)
Barbosa, 2009[25]	Dental caries	WHO criteria: 3 gr:<br>gr I: dmft/DMFT = 0<br>gr II: dmft/DMFT = 1-2<br>gr III: dmft/DMFT = 3	Clear gradient in mean and median of overall FIS scores and in the 4 subscales were observed across dental caries categories, but not statistically significant ($p > .05$, Mann-Whitney U test).
	Malocclusion	DAI: 4gr:<br>gr I: none-minor<br>gr II: definitive<br>gr III: severe<br>gr IV: handicapping	Significant differences in overall and parental emotions scores were found between minor and severe/handicapping malocclusion groups
McGrath,2007[26]	Diagnostic groups (2 groups)	Treatment required: 2 gr.<br>gr I: Paedodontic<br>gr II: Orthodontic	The mean overall FIS and subscale PE in orthodontic group were higher than in the paedodontic group ($p < .01$ and $p < .05$, respectively, Mann-Whitney U test).
Pipovic, 2024[27]	Dental caries	WHO criteria: 2 gr.<br>gr I: Caries free<br>gr II: Caries experience	Overall mean FIS-14, mean FIS-8, mean score in PA and PE subscales were significantly higher in children with caries than without caries ($p < 0.05$, student t test).
	Malocclusion	IOTN DHC: 2gr:<br>gr I: IOTN ≤ 2<br>gr II: IOTN ≥ 3	Overall mean FIS-14, mean FIS-8, mean score in PA and PE subscales were significantly higher in children with IOTN DHC ≥ 3 than IOTN DHC ≤2 ($p < .05$, student t test).
Purohit, 2021[28]	Dental caries	WHO criteria: 3 gr:<br>gr I: DMFT=0<br>gr II: DMFT=1-2<br>gr III: DMFT=3-5	Significant difference in mean FIS scores among 3 gr. ($p<.05$, one-way ANOVA). DMFT score was a significant independent predictor of total FIS score after adjusting for global oral health rating, socioeconomic status, and other covariates ($R^2=0.138$ from linear regression). DMFT increased odds of reported family impact (OR=1.37, 95% CI: 1.08–2.33, $p < .05$, logistic regression).

Study	Condition Evaluated	Hypothesis testing (e.g. group Comparison)	Main Findings (Statistical Test Used)
Purohit, 2021[28] (continued)	Oral hygiene status	Debris index (DI), Calculus index (CI), and Simplified oral hygiene index (OHI-S): each index had 3 gr:<br>gr I: good<br>gr II: fair<br>gr III: poor	Poorer oral hygiene status (DI, CI, OHI-s) was significantly associated with higher family impact scores ($p < .05$, one-way ANOVA). Oral hygiene rating (good vs. poor) was a significant predictor of FIS in linear regression. Poor oral hygiene increased odds of family impact (OR=2.99, 95% CI: 1.52–5.89, $p < .001$, logistic regression)
Vinayagamoorthy, 2020[29]	Dental caries	WHO criteria (58.2% had dental caries); 2 gr:<br>gr I: caries<br>gr II: non-caries	No statistically significant difference in mean FIS scores between 2 gr. ($p = 0.86$, type of statistics not specified).
	Malocclusion	DAI criteria (59.9% had malocclusion); 2 gr:<br>gr I: malocclusion<br>gr II: normal occlusion	Mean overall FIS scores and all subscale scores were significantly higher in the malocclusion gr. than in the normal gr. ($p < 0.001$, Mann-Whitney U test).<br>FIS scores were significantly higher in children with malocclusion (adjusted IRR = 1.86, 95% CI: 1.60–2.17, $p < .001$, negative binomial regression).
Kumar, 2016[30]	Dental caries	WHO criteria; 3 gr:<br>gr I: DMFT = 0<br>gr II: DMFT = 1-3<br>gr III: DMFT > 3	No significant difference in median FIS scores among 3 gr. ($p > .05$, Mann-Whitney U test).
	Malocclusion	Orthodontic treatment required; yes vs. no	Significant difference in median FIS scores between 2 gr. ($p < .001$, Mann-Whitney test).
	Fluorosis	Dean's index; 2 gr,<br>gr I: none to mild<br>gr II: moderate to severe	Significant difference in median FIS scores between 2 gr. ($p < .001$, Mann-Whitney U test).

Study	Condition Evaluated	Hypothesis testing (e.g. group Comparison)	Main Findings (Statistical Test Used)
Abanto, 2015[31]	Dental caries	WHO criteria (54% had dental caries); 2 gr:<br>gr I: DMFT = 0<br>gr II: DMFT ≥ 1	Median FIS scores were higher in the caries gr. for total and all subscales ($p < .001$, Mann-Whitney U test); mean scores were also higher in the caries gr. (not statistically compared).
	Malocclusion	DAI criteria (74% had malocclusion); 2 gr:<br>gr I: with malocclusion<br>gr II: without malocclusion	Median FIS scores were higher in the malocclusion gr. for total and all subscales ($p < .001$, Mann-Whitney U test); mean scores were also higher in the malocclusion gr. (not statistically compared).

FIS = Family Impact Scale; NA = not assessed; gr = group; SD = standard deviation; *r* = Spearman's rank correlation coefficient; DMFT = decayed, missing, and filled teeth; IOTN = Index of Orthodontic Treatment Need; DHC = Dental Health Component; NR = not reported; WHO = World Health Organization; DAI = Dental Aesthetic Index; PA = Parental Activity; PE = Parental Emotion; R^2 = Coefficient of Determination; OR = Odds Ratio; CI = Confidence Interval; IRR = incidence rate ratio
